# Caregiving, care burden and awareness of caregivers and patients with dementia in Asian locations: a secondary analysis

**DOI:** 10.1186/s12877-021-02178-x

**Published:** 2021-04-07

**Authors:** Yong S. Shim, Kee Hyung Park, Christopher Chen, Jacqueline C. Dominguez, Kyunghun Kang, Hee-Jin Kim, Zhen Hong, Yu-Te Lin, Leung-Wing Chu, San Jung, SangYun Kim

**Affiliations:** 1 Department of Neurology, Eunpyeong St. Mary’s Hospital, College of Medicine, The Catholic University of Korea, Seoul, Republic of Korea; 2 Department of Neurology, College of Medicine, Gachon University Gil Hospital, Incheon, Republic of Korea; 3 Department of Pharmacology, Yong Loo Lin School of Medicine, National University of Singapore, Singapore, Singapore; 4 Institute for Neurosciences, St. Luke’s Medical Center, Taguig, Philippines; 5 Department of Neurology, School of Medicine, Kyungpook National University, Daegu, Republic of Korea; 6 Department of Neurology, College of Medicine, Hanyang University, Seoul, Republic of Korea; 7 Huashan Hospital Affiliated to Fudan University, Shanghai, China; 8 Kaohsiung Veterans General Hospital, Kaohsiung, Taiwan; 9 Queen Mary Hospital, Department of Medicine, The University of Hong Kong, Pok Fu Lam, Hong Kong; 10 Department of Neurology, Hallym University Kangnam Sacred Heart Hospital, Seoul, Republic of Korea; 11 Department of Neurology, Seoul National University College of Medicine & Clinical Neuroscience Center, Seoul National University Bundang Hospital, 582, Gumi-ro 173 Beon-gil, Bundang-gu, Seongnam-si, Gyeonggi-do, 13620 Seoul, Republic of Korea

**Keywords:** Alzheimer’s disease, Asia, Burden, Caregivers, Dementia, Awareness

## Abstract

**Background:**

This study investigated the differences in caregiver activity, caregiver burden, and awareness of both caregivers and patients with Alzheimer’s disease (AD) across different Asian locations.

**Methods:**

This was a secondary analysis of a multi-national cohort study that aimed to assess caregiver activity and caregiver burden using the Caregiver Activity Scale (CAS) and Zarit Burden Interview (ZBI), respectively. Patients’ awareness of their dementia diagnosis was assessed by asking the following yes/no question: “Do you have dementia?” Caregivers’ awareness of the patient’s dementia diagnosis was assessed by asking the following yes/no question: “Does your patient have dementia?”

**Results:**

In total, 524 caregivers of patients with AD from China, Hong Kong, South Korea, the Philippines, Singapore, Thailand, and Taiwan participated. The CAS and ZBI score were significantly different across most locations (*p* < 0.001 and *p* = 0.033, respectively). Overall, 56.6% of caregivers and 37.5% of patients had awareness of the dementia diagnosis, and the proportion of patients and caregivers with awareness were also different between each location (all, *p* < 0.001).

**Conclusions:**

Caregiving, caregiver burden, and the awareness of caregivers and patients were different across many Asian locations. With understanding of cultural differences, further public education on dementia could help increase the awareness of patients and caregivers and reduce caregiver burden.

**Trial registration:**

ClinicalTrials.gov, NCT02262975. Registered 13 October 2014,

## Background

According to the World Alzheimer Report 2015, dementia currently affects more than 46 million people worldwide and this number is estimated to increase to 131.5 million by 2050 [1]. In comparison to 2009 estimates, the estimated prevalence of dementia has increased in Asia and Africa but decreased in Europe and in the United States [1]. In 2005, the Delphi Consensus Report estimated that 48% of dementia patients live in Asia and that this percentage is estimated to grow to 59% by 2050 [2]. Therefore, countries in Asia face a greater proportion of the global burden.

The World Alzheimer Report also reported that the majority of patients with dementia are still living at home, and that 94% of people living with dementia in low- and middle-income countries are cared for at home [1]. Therefore, those often directly involved in the care of dementia patients are their families, and as a result, dementia has a strong impact on the wellbeing, functioning and quality of life of not only home-dwelling dementia patients but also their family caregivers [1]. Efforts should be made to determine what factors affect caregiving activity and caregiver burden and what measures are necessary to reduce them.

A large proportion of patients with Alzheimer’s disease (AD) have anosognosia (i.e., denial, impaired awareness or a lack of awareness of their cognitive deficits, functional limitations, and disease-related behavioral changes) [3–5]. The prevalence of anosognosia in AD varies between 20 and 80%, although this is dependent on factors such as the assessment method used, sample characteristics, and the severity of dementia [5]. In addition to the poor awareness of patients themselves, the awareness of caregivers can also affect the prognosis of patients with dementia as well as caregiver burden [6]. For example, Boise et al. found that caregivers delayed seeking a diagnosis because they lacked information about dementia; caregivers believed symptoms were signs of normal aging [7]. Caregivers were also generally overwhelmed by the situation and did not know which physician to ask [7]. Therefore, poor awareness of both the patient and caregiver may significantly affect the prognosis and caregiving of dementia patients.

Caregiver burden, caregiver awareness, and patient awareness are also influenced by cultural and socioeconomic factors [8, 9]. In Asia, there is a cultural diversity that is likely to significantly affect caregiver burden and the awareness of dementia patients and their family caregivers. During the original study, the Caregiver Activity Scale (CAS), the Zarit Burden Interview (ZBI), and simple questions about awareness of the disease were conducted at baseline. A secondary analysis of the results of these surveys was conducted to investigate caregiver burden, caregiver awareness, and patient awareness in seven Asian locations, and whether cultural diversity had any effect.

## Methods

### Original study design and cohort

The original cohort study was a multi-national trial to identify treatment discontinuation rates in de novo Alzheimer’s disease patients who have been newly prescribed with DOnepezil in ASia (ADOS) (NCT02262975). In brief, the original study was conducted on patients who met the National Institute of Neurological and Communicative Disorders and Stroke and the Alzheimer’s Disease and Related Disorders Association criteria [10] for probable AD and were aged between 50 and 90 years. Inclusion criteria included patients who were prescribed donepezil monotherapy for the first time and who had a caregiver who could accompany them to the hospital. In total, 527 patients from 38 institutions across seven countries in Asia (see Appendix) were included in the original study. Among them, three caregivers did not assess the caregiver questionnaire. Therefore, in this analysis, data from 524 caregivers were analyzed.

The original study was performed between July 2014 and August 2016 and conducted in accordance with Good Clinical Practice (GCP) and the Declaration of Helsinki as well as with the regulations of the International Conference on Harmonisation. The Institutional Review Board guidelines of each center were adhered to in all research centers, and written informed consent was obtained from all patients and caregivers who participated in the study.

### Survey assessments

In the current analysis we investigated caregivers and patients with AD dementia from seven Asian geographic locations (China, Hong Kong, the Philippines, Singapore, South Korea, Taiwan, and Thailand). General demographic and background characteristics including caregiving activities, social support, and social activities of the caregiver were recorded at the first visit. The clinical characteristics of patients were also recorded including a mini-mental state exam (MMSE) score [11] and clinical dementia rating (CDR) [12].

This analysis used the CAS [13] and the ZBI [14] to assess caregivers of patients who participated in the ADOS study. The ZBI is one of the most commonly used scales for assessing the burden of caregivers who provide care for dementia patients, with a higher score indicating a greater burden [15]. The ZBI is a 22-item questionnaire measuring subjective burden, which has demonstrated high consistency and validity. The CAS was used to collect information regarding the time caregivers spent providing care during a typical 24-h period, with increased time indicative of greater burden. The clinical characteristics of the patients and caregivers were then used to determine the presence of any national or cultural differences in CAS and ZBI survey data. Survey information was collected at baseline and was not affected by whether or not participants completed a follow-up visit.

Additionally, patients’ awareness of their dementia diagnosis was assessed by asking the following yes/no question: “Do you have dementia?” Caregivers’ awareness of the patient’s dementia diagnosis was assessed by asking the following yes/no question: “Does your patient have dementia?” The CAS and ZBI scores and patient and caregiver awareness were compared between Asian locations to determine which demographic and clinical characteristics of the caregivers and patients affected caregiver burden. These assessments were performed before the initial prescription of donepezil.

### Statistical analyses

A two-sample t-test and an analysis of variance (ANOVA) were used as parametric hypothesis tests while analyzing differences between two and three groups, respectively. For non-parametric hypothesis testing, a Wilcoxon’s rank sum test and a Kruskal–Wallis test were used to test differences between two and three or more groups, respectively. A Fisher’s exact test with Monte Carlo simulation and Pearson’s Chi-square test (χ2) were used to compare percentages. To analyze the association between patients, caregiver awareness, ZBI, and CDR scores, linear regression and logistic regression methods were used. The statistical significance threshold was set to α = 0.05 with a 95% confidence level. Statistical analyses were performed using SAS® software (version 9.3; SAS Institute Inc., Cary, NC, USA).

## Results

### Background characteristics of caregivers and patients

In the original study, 524 caregivers of patients with AD were recruited from China (*n* = 101), Hong Kong (n = 10), South Korea (*n* = 259), the Philippines (*n* = 23), Singapore (*n* = 52), Thailand (*n* = 6), and Taiwan (*n* = 73) (Table 1). The overall mean age of caregivers was 59.17 ± 15.35 years with the oldest age in Hong Kong (80.20 ± 6.88 years) and the lowest age in the Philippines (46.30 ± 14.23 years). In total, 61.3% of caregivers were female; however, there were more male caregivers in China (53.5%) and Taiwan (52.1%). The mean duration of education of caregivers was 11.58 ± 5.20 years, with longer durations of education in the Philippines (15.17 ± 2.87 years) and shorter durations of education in Hong Kong (3.00 ± 4.55 years) and Thailand (3.00 ± 1.73 years). There were significant differences across all seven locations for mean age, sex, marriage status, duration of education, employment status, and relationship to the patient (all, *p* < 0.001). In general, caregivers were the spouse (35.3%), daughter (29.4%), or son (21.5%). The percentage of children as caregivers was 58.9%, if son, daughter, or son- or daughter-in-law were included; however, there were significant differences between locations (*p* = 0.005).

**Table 1:** Demographic and clinical characteristics of caregivers

	Total (*n* = 524)	South Korea (*n* = 259)	China (*n* = 101)	Taiwan (*n* = 73)	Singapore (n = 52)	Philippines (n = 23)	Hong Kong (n = 10)	Thailand (*n* = 6)	*p*-value
Age, years	59.17 ± 15.35 (*n* = 514)	60.09 ± 14.76 (*n* = 254)	61.64 ± 16.64 (*n* = 99)	57.44 ± 13.90 (*n* = 70)	54.21 ± 12.51	46.30 ± 14.23	80.20 ± 6.88	56.67 ± 19.59	< 0.001
Sex (male:female)	203:321	82:177	54:47	38:35	20:32	4:19	4:6	1:5	< 0.001
Married	467/521	237/258	97/99	10/63	41/52	17/23	10/10	2/6	< 0.001
Duration of education, years	11.58 ± 5.20 (*n* = 505)	10.72 ± 5.38 (n = 247)	12.14 ± 3.87 (*n* = 97)	13.15 ± 5.30	13.02 ± 4.22	15.17 ± 2.87	3.00 ± 4.55	3.00 ± 1.73 (*n* = 3)	< 0.001
Employed	232/512	108/256	36/93	33/73	35/51	15/23	1/10	4/6	0.001
Caregiver relationship to the patient									0.005
Spouse	184	105	34	24	12	4	4	1	
Daughter-in-law	38	25	6	4	2	0	1	0	
Son	112	45	26	23	13	3	1	1	
Daughter	153	71	28	18	21	9	3	3	
Son-in-law	4	2	0	1	0	0	1	0	
Other	30	10	5	3	4	7	0	1	
Caregiver and patient cohabiting	324/521	148/258	66/99	52/73	31/52	19/23	5/10	3/6	0.069

Data are presented as n or mean ± standard deviation

Caregiver relationship to the patient was provided by only 258 caregivers in South Korea and 99 caregivers in China

Table 2 describes the demographic and clinical characteristics of patients. Overall, patients mean age was 75.68 ± 7.07 years. The mean MMSE score was 18.66 ± 5.45 (*n* = 504), and the mean CDR (global) was 0.94 ± 0.53 (*n* = 522; range 0.5–3) in the overall population.

**Table 2:** Demographic and clinical characteristics of patients with dementia

	Total	South Korea	China	Taiwan	Singapore	Philippines	Hong Kong	Thailand	*p*-value
	(*n* = 524)	(*n* = 259)	(*n* = 101)	(*n* = 73)	(*n* = 52)	(*n* = 23)	(*n* = 10)	(*n* = 6)	
Age, years	75.68 ± 7.07	75.21 ± 6.89	74.61 ± 7.26	76.85 ± 7.42	75.65 ± 7.19	78.52 ± 4.52	80.20 ± 6.88	81.17 ± 8.04	0.01
Sex (male:female)	203:321	117:142	36:65	20:53	15:37	10:13	4:6	1:5	0.053
Duration of education, years	7.22 ± 5.33	6.36 ± 4.83	9.87 ± 5.37	6.33 ± 4.63	5.13 ± 4.75	14.39 ± 3.94	3.20 ± 4.96	8.00 ± 6.23	< 0.001
MMSE score	18.66 ± 5.45 (*n* = 504)	19.93 ± 4.73 (*n* = 255)	18.16 ± 6.25 (*n* = 99)	16.41 ± 5.23 (*n* = 73)	17.00 ± 5.07 (*n* = 51)	17.96 ± 6.83 (*n* = 23)		14.67 ± 8.39 (*n* = 3)	< 0.001
CDR (range)	0.94 ± 0.53 (0.5–3)	0.84 ± 0.44 (0.5–3)	1.07 ± 0.66 (0.5–3)	0.97 ± 0.51 (0.5–2)	1.10 ± 0.52 (0.5–3)	1.11 ± 0.58 (0.5–2)	0.95 ± 0.60 (0.5–2)	0.67 ± 0.26 (0.5–1)	< 0.001
CDR-SOB	5.26 ± 3.17 (n = 522)	4.61 ± 2.67 (*n* = 257)	6.35 ± 3.73 (n = 101)	5.32 ± 3.28 (n = 73)	6.16 ± 3.27 (n = 52)	6.13 ± 3.52 (n = 23)	5.15 ± 3.28 (*n* = 10)	3.25 ± 1.13 (n = 6)	< 0.001

Data are presented as n or mean ± standard deviation

*CDR* Clinical dementia rating, *CDR-SOB* Clinical dementia rating-sum of boxes, *MMSE* Mini-mental state exam

### Caregiver activity scale and Zarit burden interview score

**Fig. 1:**
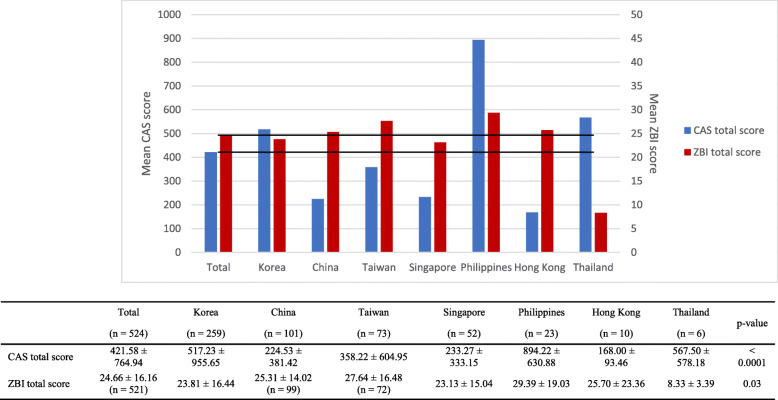
Mean caregiver activity scale (CAS) and Zarit burden interview (ZBI) scores

Figure 1 illustrates the CAS and ZBI scores obtained in each location. The CAS score (*p* < 0.001) and ZBI score (*p* = 0.033) were significantly different between each location.

Linear regression analysis showed that in all studied locations, the CAS score was generally higher for patients who were male, overweight, and/or tall and who were cared for by an older caregiver, unemployed caregiver, poorly educated caregiver, non-child caregiver, or a caregiver who resided with them (Table 3). Additionally, in China, the CAS score was high if the patient had a severe CDR (*n* = 96; estimate 137.96 ± 58.37, *p* = 0.02). In Singapore, the CAS score was high if the caregiver was unmarried (*n* = 49; estimate 392.76 ± 110.21, *p* = 0.001), or if the patient was aware of their illness (n = 49; estimate 209.15 ± 93.88, *p* = 0.031). In the Philippines, the CAS score was higher according to the lower MMSE score (*n* = 23; estimate − 62.60 ± 14.81, *p* < 0.001) and the more severe CDR (*n* = 23; estimate 678.90 ± 183.87, *p* = 0.001) of the patients.

**Table 3:** Caregiver activity scale (CAS) and Zarit burden interview (ZBI) scores

	CAS				ZBI			
Variables	n	Estimate	Standard error	*p*-value	n	Estimate	Standard error	*p*-value
Patient’s sex (female)	495	− 387.0084	68.662	< 0.0001	521	−3.3414	1.448	0.021
Patient’s age	495	4.8481	4.824	0.3154	521	0.2237	0.1	0.026
Patient’s duration of education	495	4.8044	6.423	0.4548	521	0.0065	0.133	0.961
Patient’s weight	450	12.4228	3.07	< 0.0001	472	0.0583	0.07	0.407
Patient’s height	449	14.5499	3.337	< 0.0001	471	0.1289	0.077	0.097
MMSE	476	9.7593	6.475	0.1324	501	−0.455	0.13	< 0.001
CDR (sum of boxes)	493	16.0826	10.847	0.1388	519	1.7492	0.21	< 0.001
CDR (global)	493	94.7484	65.635	0.1495	519	9.9107	1.275	< 0.001
Patient’s awareness (none)	487	117.4496	71.767	0.1024	508	1.0642	1.469	0.469
Caregiver’s sex (female)	495	131.9129	70.314	0.0612	521	3.5599	1.447	0.014
Caregiver’s age	486	6.8393	2.24	0.0024	511	0.0415	0.046	0.370
Caregiver’s duration of education	484	−29.3388	6.587	< 0.0001	503	−0.0846	0.138	0.541
Caregiver’s occupation (none)	484	226.7616	69.114	0.0011	509	2.1644	1.435	0.132
Caregiver’s marital status (single)	494	66.7254	113.298	0.5562	518	−1.0485	2.326	0.652
Caregiver’s awareness (none)	494	36.5177	69.646	0.6003	517	−6.3056	1.41	< 0.001
Relationship with caregiver (child)	426	− 431.9034	75.527	< 0.0001	448	−1.6973	1.516	0.267
Cohabitation (no)	493	− 357.2101	69.206	< 0.0001	519	−5.4699	1.446	< 0.001

*CDR* Clinical dementia rating, *MMSE* Mini-mental state exam

Generally, in all studied locations, the ZBI score was high if the patient was male or old, with a low MMSE score and a high CDR, and if the caregiver was female or cohabiting with the patient (Table 3). The patient’s awareness of the illness was unrelated, but the ZBI score was high if the caregiver was aware of the patient’s dementia. Specifically, in South Korea, the ZBI score was high if the caregiver’s duration of education was short (*n* = 247; estimate − 0.51 ± 0.19, *p* = 0.008), and the caregiver was unemployed (*n* = 256; estimate 4.71 ± 2.05, *p* = 0.023). In Singapore, the ZBI score was high if the patient was unaware of the illness (*n* = 52; estimate 16.08 ± 3.61, *p* < 0.001). In Hong Kong, the ZBI score was high if the caregiver was spouse of the patients, not the child (*n* = 8; estimate − 33.50 ± 12.58, *p* = 0.037).

### Awareness of caregivers and patients

**Fig. 2:**
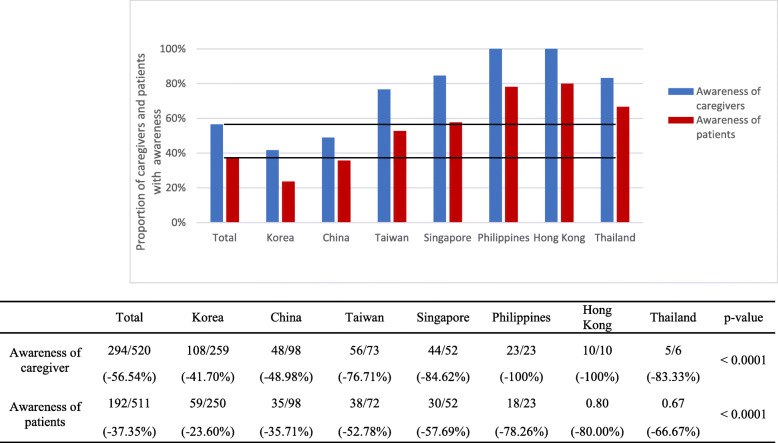
Proportion of caregivers and patients with awareness

The awareness of patients and caregivers of the patients’ dementia diagnosis differed by location (Fig. 2). Overall, 56.5% of caregivers and 37.4% of patients had awareness; however, in Hong Kong and the Philippines, 100.0% of caregivers had awareness. In contrast, 49.0 and 41.7% of caregivers in China and South Korea had awareness, respectively. The proportion of patients with awareness were 80.0 and 78.3% in Hong Kong and the Philippines, respectively, followed by Thailand (66.7%), Singapore (57.7%), Taiwan (52.8%), China (35.7%), and South Korea (23.6%), in descending order (*p* < 0.001).

Logistic regression analysis showed that, in general, patients were likely to have awareness if they were female and if their caregiver was young, educated or their child (Table 4). Furthermore, patients had awareness if their caregiver also had awareness. Specifically, in Taiwan and Singapore, patients had awareness if there was a high MMSE score (*n* = 73; estimate 0.10 ± 0.05, odds ratio [OR] 1.102 [95% confidence interval (CI): 1.002–1.213, *p* = 0.046] and *n* = 51; estimate ± SE, 0.22 ± 0.08, OR 1.245 [95% CI: 1.064–1.456, *p* = 0.006], respectively), and not so severe CDR_SOB (n = 73; estimate − 0.17 ± 0.08, OR 0.847 [95% CI: 0.726–0.988, *p* = 0.035 and *n* = 52; estimate − 0.44 ± 0.16, OR 0.642 [95% CI: 0.470–0.877, *p* = 0.005], respectively). In addition, in Singapore, the lower the CDR (n = 52; estimate − 2.28 ± 0.85, OR 0.102 [95% CI: 0.019–0.543, *p* = 0.007]) and the lower the ZBI (n = 52; estimate − 0.09 ± 0.26, OR 0.915 [95% CI: 0.870–0.962, *p* = 0.001]), the more likely the patients were to have awareness. In China and South Korea, patients’ awareness was inversely proportional to caregivers’ awareness (*n* = 98; estimate − 0.88 ± 0.43, OR 0.415 [95% CI: 0.178–0.971, *p* = 0.042] and *n* = 258; estimate − 2.06 ± 0.35, OR 0.128 [95% CI: 0.064–0.254, *p* < 0.001], respectively).

**Table 4:** Factors that influence the awareness of caregivers and patients

Variables	Patient awareness						Caregiver awareness					
	n	Estimate	Standard	Odds	Odds Ratio	*p*-value	n	Estimate	Standard	Odds	Odds Ratio	*p*-value
			Error	Ratio	[95% CI]				Error	Ratio	[95% CI]	
Patient’s sex (female)	524	0.4008	0.1897	1.493	[1.029,2.165]	0.0346	524	0.4205	0.1808	1.523	[1.068,2.170]	0.02
Patient’s age	524	−0.0097	0.0128	0.99	[0.966,1.016]	0.4486	524	0.0429	0.0128	1.044	[1.018,1.070]	< 0.001
Patient’s duration of education	524	0.0119	0.017	1.012	[0.979,1.046]	0.4842	524	−0.0195	0.0165	0.981	[0.949,1.013]	0.237
MMSE	504	0.0146	0.0172	1.015	[0.981,1.050]	0.3981	504	−0.1166	0.0187	0.89	[0.858,0.923]	< 0.001
CDR-SOB	522	−0.0508	0.0299	0.95	[0.896,1.008]	0.0891	522	0.2566	0.0379	1.292	[1.200,1.392]	< 0.001
CDR (global)	522	−0.2392	0.1787	0.787	[0.555,1.118]	0.1808	522	1.3555	0.2243	3.879	[2.499,6.021]	< 0.001
Patient’s awareness (no)							511	−1.7293	0.2152	0.177	[0.116,0.270]	< 0.001
Caregiver’s sex (female)	524	0.1176	0.1869	1.125	[0.780,1.622]	0.5292	524	−0.036	0.1808	0.965	[0.677,1.375]	0.842
Caregiver’s age	514	−0.0172	0.0061	0.983	[0.971,0.995]	0.0045	514	−0.0136	0.0059	0.986	[0.975,0.998]	0.020
Caregiver’s duration of education	505	0.0543	0.0185	1.056	[1.018,1.095]	0.0033	505	0.0373	0.0175	1.038	[1.003,1.074]	0.032
Caregiver’s occupation (none)	512	−0.0926	0.1844	0.912	[0.635,1.309]	0.6158	512	−0.2444	0.1798	0.783	[0.551,1.114]	0.174
Marital status of caregiver (single)	521	0.5398	0.2891	1.716	[0.974,3.023]	0.0619	521	0.4911	0.3033	1.634	[0.902,2.961]	0.105
Relationship with caregiver (child)	449	0.6964	0.2052	2.006	[1.342,3.000]	0.0007	449	0.6053	0.1943	1.832	[1.252,2.681]	0.002
Cohabitation (no)	521	−0.0561	0.1876	0.945	[0.655,1.366]	0.765	521	0.0402	0.1822	1.041	[0.728,1.488]	0.826
Primary caregiver (no)	515	0.0569	0.2012	1.059	[0.714,1.570]	0.7772	515	0.064	0.1967	1.066	[0.725,1.568]	0.745
CAS	495	−0.0002	0.0001	1	[0.999,1.000]	0.1358	495	−0.0001	0.0001	1	[1.000,1.000]	0.615
ZBI	521	−0.004	0.0057	0.996	[0.985,1.007]	0.4787	521	0.0252	0.0059	1.026	[1.014,1.037]	< 0.001
Caregiver’s awareness (no)	520	−1.7397	0.2144	0.176	[0.115,0.267]	< 0.0001						

*CAS* Caregiver activity scale, *CDR* Clinical dementia rating, *CDR-SOB* Clinical dementia rating-sum of boxes, *CI* Confidence interval, *MMSE* Mini-mental state exam, *ZBI* Zarit burden interview

Caregivers were more likely to have awareness if the patient was female, older, and severely demented and if the caregiver was young, had a longer duration of education, and had a high ZBI score (Table 4). Caregivers’ awareness was inversely proportional to patients’ awareness (Table 5), and caregivers were more likely to have awareness if they were the patient’s children rather than spouse. Specifically, in China, caregivers’ awareness was high if the CDR was high (*n* = 101; estimate ± SE, 1.47 ± 0.39, OR 4.329 [95% CI: 2.005–9.349, *p* < 0.001]), and this was inversely proportional to patients’ awareness (*n* = 98; estimate − 0.88 ± 0.43, OR 0.415 [95% CI: 0.178–0.971, *p* = 0.043]).

**Table 5:** Lack of awareness stratified by baseline clinical dementia rating (CDR)

	Baseline CDR 0.5	Baseline CDR 1	Baseline CDR 2	Baseline CDR 3	*p*-value
	*n* (%)	*n* (%)	*n* (%)	*n* (%)	
*n*	160	188	41	4	< 0.0001
Yes	69 (43.13)	118 (62.77)	37 (90.24)	4 (100.00)	
No	91 (56.88)	70 (37.23)	4 (9.76)	0 (0.00)	

## Discussion

The present study showed that 56.6% of caregivers had awareness of their patient’s dementia and 37.4% of patients were aware of their dementia, and that patients’ and caregivers’ awareness were mutually influential. Patients were more likely to be aware of their dementia if they were female and the caregiver was young, highly educated, and one of the patient’s children. From the caregivers’ perspective, high awareness was achieved if they were young, female, highly educated, and one of the patient’s children, and if the patient was highly demented.

The CAS score was more likely to be high if the patient was male, overweight, and tall and if the caregiver was old, poorly educated, unemployed, the spouse, the primary caregiver, or cohabiting with the patient. The ZBI score was more likely to be high if the patient was male, old, severely demented, or with dysfunctional cognitive skills and if the caregiver was female, cohabiting with the patient, the primary caregiver, and if the caregiver was aware of the patient’s illness. Interestingly, patients’ awareness of their illness did not significantly influence caregiver CAS and ZBI scores; however, the ZBI score was high when the caregiver was aware of the patient’s illness.

The factors that influence CAS and ZBI scores were similar to what has been previously published in other studies. Caregivers who are of an advanced age [16, 17], are female [18–20], and are cohabiting with the patient, are more likely to experience a greater burden [21]. Several studies have also reported that spousal caregivers experience the highest level of burden [16, 21, 22]. Furthermore, some studies have also suggested that moderate-to-severe disability, which affects a patient’s ability to perform basic daily activities, is related to a higher caregiver burden [16, 21, 23, 24].

However, care burden about dementia patients in Asia is somewhat different, compared to the Western countries. Ikels followed a sample of 200 elders from 1987 to 1991 in Canton, China and reported that the experience of giving care to patients with dementia was psychologically less threatening than for people in the United States [25, 26]. This is possibly because in China there are cultural concepts that preserve the patients’ sense of self for longer and caregiving offers a greater intrinsic reward to the family caregiver.

Caregiver burden has previously been defined as “a multidimensional response to physical, psychological, emotional, social, and financial stressors associated with the caregiving experience” [27]. This description differs from caregiving, which is referred to as the “activities and experiences involved in providing help and assistance to relatives who are unable to provide for themselves”, which does not include the psychological distress that may come from the act of caregiving [28]. In this study we used both CAS and ZBI to distinguish caregiving activity from caregiver burden in each location.

The factors influencing awareness also differed by location. The patients’ and caregivers’ awareness were low, below 50%, in South Korea and China compared to other locations. This is presumably because South Korea and China have a relatively more traditional Confucian culture, which might prevent dementia from being recognized as an illness [29]. In previous studies, there have been reports that there is a paucity of information on dementia in South Korea and China [30, 31]. One longitudinal prospective study of cognitive impairment among elderly adults showed that the prevalence rate of dementia in persons aged 65 years and older was 4.6% in Shanghai [32]. This is much lower than reports from Western countries where researchers have reported difficulty in recruiting Asian caregivers who perceive a sense of shame for having a relative with AD [33–35]. Furthermore, Asian caregivers have been shown to delay seeking an AD diagnosis because they lacked information about dementia and believed the symptoms were signs of normal aging [7]. A large proportion of patients with AD have anosognosia [5, 6], and in our study the greater the severity of dementia the more their awareness deteriorated. With higher MMSE scores observed in South Korea and China, it might also be considered that anti-dementia medications could be prescribed earlier before patients and their family recognize their cognitive dysfunction. Interestingly, in this study, the awareness in patients was 20–30% lower than the awareness in caregivers, even though each country has differences in culture and education. When exploring the emotional impact of an Alzheimer’s disease diagnosis, although the majority of patients display signs of an emotional crisis, a range of responses including lack of insight, active denial, and positive coping responses have been reported [36]. In addition, a lack of awareness of cognitive functioning deficits is a complex and multidimensional phenomenon that is also related to a lack of awareness of functional activity impairments, age, and caregiver burden [37]. Further analysis of our findings would be helpful for understanding and developing strategies to lessen caregiver burden in different Asian countries.

A caregiver’s awareness is strongly influenced by personal preferences and practicality does not seem to play a substantive role when making a decision related to caregiving. Strong family-related emotions can also often overwhelm rational decision-making, and therefore, in the case of dementia, medical professionals are required to play a more explicit role in the decision-making process by anticipating transitions in care and outlining options for care and treatment in advance. The assessment of awareness has a high clinical relevance in cases of dementia, particularly when considering the impact this has on patients and their family as well as the social implications of their increased need for medical, social, legal, and financial support [38, 39].

One of the limitations of this study was the differences in both patient and caregiver sample sizes in each country, which is likely to affect the accuracy of any national comparisons. In particular, the number of South Korean patients was considerably higher than that of other locations, which may have influenced our findings. In addition, the differences in sample sizes from each country could be due to differences in refusal rate, which could impart selection bias; however, we were unable to analyze the refusal rate because of differences between the sample sizes of each country. Finally, although we aimed to assess awareness of dementia before the initial prescription of donepezil, it is possible that some patients were diagnosed with dementia and provided with information on their disease before our assessment. Therefore, these patients are likely to have a higher level of awareness of their dementia diagnosis at baseline.

## Conclusions

This study examined factors that affect caregiver burden as well as patient and caregiver awareness of dementia across seven Asian locations. This analysis offers a unique perspective on patients with dementia and caregiver management. Ultimately, these data may be useful when planning future multinational studies by incorporating the location and cultural diversity that can influence the care of patients with dementia.

## Data Availability

The datasets used and/or analyzed during the current study are available from the corresponding author upon reasonable request.

## Appendix

The following 38 institutions participated in this study: Seoul National University Bundang Hospital, Republic of Korea; Bucheon St. Mary’s Hospital, Republic of Korea; Gachon Gil Medical Center, Republic of Korea; Hanyang University Seoul Hospital, Republic of Korea; Korea University Anam Hospital, Republic of Korea; Pusan National University Hospital, Republic of Korea; Eulji University Hospital, Republic of Korea; Ewha Womans University Medical Center, Republic of Korea; Inha University Hospital, Republic of Korea; Chonbuk National University Hospital, Republic of Korea; Kyungpook National University Medical Center, Republic of Korea; VHS Medical Center, Republic of Korea; Hallym University Medical Center, Kangnam Sacred Heart Hospital, Republic of Korea; Kangwon National University Hospital, Republic of Korea; First Affiliated Hospital of Medical College of Xi’an Jiaotong University, China; Shanghai Huashan Hospital, affiliated to Fudan University, China; Peking Union Medical College Hospital, China; Peking University Sixth Hospital, China; Renji Hospital Affiliated to Shanghai Jiao Tong University School of Medicine, China; Nanjing Drum Tower Hospital, China; Peking University Third Hospital, China; Changhua Christian Hospital, Taiwan; Chi Mei Hospital, Liouying, Taiwan; Kaohsiung Medical University Chung-Ho Memorial Hospital, Taiwan; Kaohsiung Veterans General Hospital, Taiwan; Taipei Medical University Shuang-Ho Hospital, Taiwan; Chi Mei Medical Center, Taiwan; Shin Kong Wu Ho-Su Memorial Hospital, Taiwan; King Chulalongkorn Memorial Hospital (KCMH), Thailand; Phramongkutklao Hospital, Thailand; Ramathibodi Hospital, Thailand; Siriraj Hospital, Thailand; National University Hospital, Singapore; National Neuroscience Institute, Singapore; St. Luke’s Medical Center, Philippines; Queen Mary Hospital, Hong Kong.

## Abbreviations

AD: Alzheimer’s disease
CAS: Caregiver activity scale
CDR: Clinical dementia rating
CI: Confidence interval
MMSE: Mini-mental state exam
NHP: Nursing home placement
OR: Odds ratio
SD: Standard deviation
SE: Standard error
ZBI: Zarit Burden Interview
